# Mycoplasma pneumoniae Outbreak in 2023: Post-pandemic Resurgence of an Atypical Bacterial Pathogen

**DOI:** 10.7759/cureus.58757

**Published:** 2024-04-22

**Authors:** Pallavi Upadhyay, Vijay Singh

**Affiliations:** 1 Research & Development (R&D), HealthTrackRX, Denton, USA

**Keywords:** mycoplasma pneumoniae, multiplex pcr, covid-19 pandemic, respiratory tract infections, community acquired pneumonia

## Abstract

The syndromic nature of infections caused by pneumonia-causing pathogens including *Mycoplasma pneumoniae *necessitates detection via multiplex PCR for accurate and timely diagnosis to control the infection spread. In this study, we demonstrate an increase in the detection of *M. pneumoniae* in the outpatient population, during 2023, as compared to the previous two years (2021-2022). In this aggregated survey, respiratory samples collected within the continental United States were tested for the presence of *M. pneumoniae* and other respiratory bacterial and viral pathogens using a multiplex PCR assay.

Patient data was analyzed on the basis of age, gender and geographical location. The positive detection of *M. pneumoniae* in 2021 and 2022 was 0.004% and 0.006%, respectively. The positivity rate of *M. pneumoniae* in 2023 increased to 0.21%. The highest proportion of *M. pneumoniae* cases were detected from Georgia with the outbreak generally concentrated in large urban settings. Median age of the patients testing positive for *M. pneumoniae* was 10 (interquartile range [IQR] 8-18) years with an almost equal distribution between male and female patients. Other respiratory, viral and bacterial, pathogens detected in samples positive for *M. pneumoniae* were similar in proportion to the *M. pneumonia-*negative population. A survey of the ICD-10 codes submitted in conjunction with the samples suggests that the current outbreak is mostly associated with upper respiratory tract infections.

The present study is the first detailed report in the United States that shows an unprecedented increase in the detection of *M. pneumoniae* in the outpatient population during 2023. Our analysis suggests that this outbreak was not associated with any other bacterial or viral respiratory pathogen. The outbreak of this atypical pathogen was concentrated in the pediatric population in large urban areas. The 2023 outbreak could be a return of the cyclical *M. pneumoniae* outbreaks witnessed prior to the COVID-19 pandemic. Our study highlights the importance of performing continuous surveillance of respiratory pathogens, especially in the altered epidemiological landscape of the post-COVID world.

## Introduction

*Mycoplasma pneumoniae* belongs to the class Mollicutes (family Mycoplasmataceae). *M. pneumoniae* are fastidious bacteria with no cell wall and thus cannot be identified via Gram stain and require unique and atypical culture methods for detection. It is known to cause respiratory infections of both the upper and lower tract, with a wide range of clinical presentations including headaches, body aches, fever, acute bronchitis, pharyngitis, and otitis. *M. pneumoniae* is considered as one of the major causes of community-acquired pneumonia (CAP), as almost all of the *M. pneumoniae* outbreak incidences have been reported in closed community or congregate settings, such as hospitals, clinics, military, residential, educational institutions, and long-term care facilities [1].

Occurrences of *M. pneumoniae* were quite common prior to the recent pandemic. According to the Centers for Disease Control and Prevention (CDC), around 2 million incidences of *M. pneumoniae* are reported annually within the United States [2]. According to a study, between 2017 and 2020, the global prevalence of *M. pneumoniae* infections was estimated at approximately 8.6% [3], which lowered down to 1.6% during the peak of the pandemic, expectedly, due to pandemic-related lockdowns, travel restrictions, and non-pharmaceutical interventions. In addition, the disruption of routine healthcare services and disease surveillance initiatives, during the pandemic, had a negative impact on the overall tracking and surveillance of respiratory tract infections (RTIs). The transmission rate plummeted further down to 0.7% from 2021 to 2022 [3]. Interestingly, during this time period, incidences of respiratory pathogens, both viral and bacterial, depicted a spike but the infection rates for *M. pneumoniae* remained low [4]. The global prospective surveillance study of *M. pneumoniae* (ESGMAC MAPS study) showed a resurgence of *M. pneumoniae* detections during April 1, and September 30, 2023, especially in Europe and Asia [5].

In the present paper, we show a more than 50-fold increase in the incidence of *M. pneumoniae* infections in 2023 as compared to the previous two years (2021-2022), especially since July 2023, in the outpatient population in the United States. In this aggregated survey of temporal, geographic and demographic distribution of the outbreak we analyzed if the *M. pneumoniae* infections were associated with co-detections of other respiratory pathogens. In addition, the hypothesis is that exclusive ICD10 diagnosis codes are associated with *M. pneumoniae* infections when compared to other respiratory pathogens. Our data demonstrates the outbreak of this respiratory pathogen in the general population and supports the continued surveillance for both regular and atypical respiratory pathogens especially in the public health scenario altered by the COVID-19 pandemic in the last few years.

## Materials and methods

In this aggregate survey, respiratory samples (nasal, oropharyngeal, and sputum swabs), collected within the continental United States, were tested at the HealthTrackRx laboratories between January 1, 2021, and December 31, 2023. Data analysis was performed only on samples that were collected in outpatient settings (urgent care centers and independent physician offices) from non-hospitalized patients symptomatic for respiratory infections and their test requisition forms included relevant ICD10 diagnosis codes. Demographic data with respect to age (distributed in increments of 10 years), gender and location (zip code and state) was collected and analyzed for the deidentified patient samples.

Nucleic acid extraction and real-time PCR

Patient swabs were suspended in the PrimeStore^TM^ molecular transport medium (Longhorn Diagnostics, Maryland, USA) and nucleic acid isolation was performed following the manufacturer’s instructions using the MagMAX^TM^ viral/pathogen nucleic acid isolation kits on the automated KingFisher^TM^ Flex Purification System (Thermo Fisher Scientific, California, USA). Real-time PCR analysis was performed using the QuantStudio 12K Flex Real-Time PCR system as per the manufacturer’s instructions (Thermo Fisher Scientific, California, USA) and as previously described [6]. PCR cycling was performed using the following program: single-step enzyme activation (95 °C) for 10 min, followed by 40 cycles of denaturation (95 °C) for 15 s, and annealing/extension (60 °C).

Patient samples were tested for the following microbial pathogens:

(1) Bacteria -- *Bordetella pertussis*, *Bordetella parapertussis*, *Chlamydophila pneumoniae*, *Moraxella catarrhalis*, *Mycoplasma pneumoniae*, *Proteus mirabilis*, *Serratia marcescens*, *Streptococcus agalactiae*, *Streptococcus pyogenes*, *Streptococcus pneumoniae*, *Staphylococcus aureus*, *Klebsiella pneumoniae*, *Legionella pneumophila* and *Haemophilus influenzae*.

(2) Viruses -- Adenovirus, Coronavirus (NL63, HKU1, 229E, OC43), Human metapneumovirus, Rhinovirus, Enterovirus, Influenza Virus (A, B), Parainfluenza Virus (1, 2, 3, 4), Respiratory Syncytial Virus (A, B), Epstein-Barr Virus, Human herpesvirus 6 and Varicella zoster Virus.

Statistical analysis

All categorical variables are expressed as numerical value (%). Due to its non-normal distribution in our data, patient age is represented as interquartile ranges (IQR). Within the *M. pneumoniae* positive and negative population, data was categorized on the basis of co-detection with other respiratory pathogens and the proportion of the various International Classification of Diseases, 10th Revision codes (ICD-10) associated with the patient samples. Chi-square analysis was used to compare the different groups. A p*-*value of less than 0.05 was considered to be indicative of a statistically significant difference. All statistical analysis was performed using R version 4.3.2 (R Foundation for Statistical Computing, Vienna, Austria).

## Results

**Figure 1:**
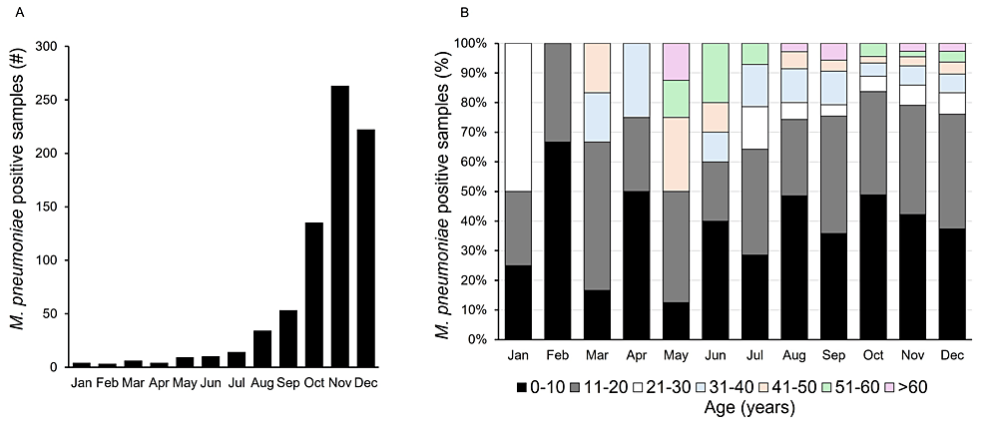
Mycoplasma pneumoniae outbreak in 2023. (A) Positive detection of *M. pneumoniae* by PCR during January – December 2023. (B) Age-wise distribution of *M. pneumoniae* positive patent samples during January – December 2023.

A total of 582,752 samples were submitted for testing at the HealthTrackRx laboratories, between January 1, 2021, and December 31, 2023. The samples were subjected to PCR testing for the presence of bacterial and viral respiratory pathogens as previously described [6]. Among the surveyed outpatient population, only 13 samples tested positive for *M. pneumoniae* between 2021 [*n* = 3/61,498 (0.004%)] and 2022 [*n* = 10/165,423 (0.006%)]. A sharp increase in the positive detection of *M. pneumoniae* was detected in 2023 with the positivity rate for the pathogen climbing to 0.21% (*n* = 757/355,831) (Figure 1A).

During 2023, the median age of the patients testing positive for *M. pneumoniae* was 10 (interquartile range [IQR] 8-18) years. Distribution of the patient population based on age showed the highest proportion of positive detections within the 0-10 years age group [*n* = 310 (41.6%)] followed by 11-20 years [*n* = 275 (36.9%)]. Month-over-month distribution of the positive detections for *M. pneumoniae* followed the same trend with the 0-10- and 11-20 years age groups comprising the majority of positive cases (Figure 1B). Classification of the positive samples based on gender showed an almost equal distribution between females [*n* = 377 (49.8%)] and males [*n* = 380 (50.1%)]. In 2023, the highest proportion of *M. pneumoniae* cases was detected from Georgia [*n *= 188 (24.8%)], followed by Ohio [*n *=156 (20.6%)], Texas [*n *= 68 (8.9%)] and California [*n *= 55 (7.2%)] (Table 1).

**Table 1:** PCR-positive detection of Mycoplasma pneumoniae by state, United States, January – December 2023

State	*M. pneumoniae* positive detections (Number)	*M. pneumoniae* positive detections (%)
Georgia	188	24.83
Ohio	156	20.61
Texas	68	8.98
California	55	7.27
Florida	39	5.15
Kentucky	34	4.49
Illinois	31	4.10
Mississippi	19	2.51
N. Carolina	18	2.38
S. Carolina	17	2.25
Colorado	15	1.98
Maryland	13	1.72
Indiana	12	1.59
Arizona	11	1.45
Massachusetts	10	1.32
Connecticut	9	1.19
Arkansas	7	0.92
Pennsylvania	6	0.79
Louisiana	5	0.66
New Mexico	5	0.66
Michigan	4	0.53
Nevada	4	0.53
Tennessee	4	0.53
Iowa	3	0.40
Minnesota	3	0.40
Virginia	3	0.40
Alabama	2	0.26
Kansas	2	0.26
Missouri	2	0.26
Nebraska	2	0.26
New Jersey	2	0.26
Oregon	2	0.26
Rhode Island	2	0.26
Washington	2	0.26
Delaware	1	0.13
Oklahoma	1	0.13

The presence of other respiratory pathogens showed the co-detection of other bacterial and viral pathogens. Pathogens co-detected in samples positive for *M. pneumoniae* were similar in proportion to the *M. pneumoniae-*negative population. The co-detected pathogens at a statistically significant difference were lower than the negative population. However, Chi-square analysis revealed no statistically significant difference in the association of other respiratory pathogens between the patient samples detected positive versus negative for *M. pneumoniae* (Table 2).

**Table 2:** Observed co-detection rates (%) of different respiratory pathogens associated with Mycoplasma pneumoniae positive and negative patient samples. * Represents statistically significant difference (p<0.05)

Virus	*M. pneumoniae *+	*M. pneumoniae* -
Adenovirus	1.74	2.60
Coronaviruses (229E, NL63, HKU1, OC43)	0.95	2.00
SARS-CoV-2	0.78*	7.90
Human metapneumovirus	0.16	0.59
Influenza Virus (A, B)	0.95*	4.10
Parainfluenza Virus (types 1,2,3,4)	1.74*	3.10
Respiratory Syncytial Virus	2.52*	5.30
Rhinovirus/ Enterovirus	13.41*	27.00
Bacteria		
Escherichia coli	0.95*	3.50
Haemophilus influenzae	19.72	19.30
*Klebsiella* *pneumoniae*	0.16*	1.30
Moraxella catarrhalis	9.78*	19.90
*Proteus* *mirabilis*	0.16	0.40
Pseudomonas aeruginosa	0.32*	1.50
Serratia marcescens	0.16	0.30
Staphylococcus aureus	9.15	9.50
Streptococcus agalactiae	1.10	1.30
Streptococcus pneumoniae	35.02	31.60
Streptococcus pyogenes	2.21	2.20

*M. pneumoniae* infections are associated with both the upper and lower respiratory tract. Our survey of the ICD-10 codes submitted in conjunction with the samples suggests that the current outbreak is mostly associated with upper RTIs. The ICD-10 codes associated with the specimens were mostly related to RTIs and their syndromic manifestations (Table 3).

**Table 3:** International Classification of Diseases, 10th revision, Clinical Modification codes associated with PCR positive Mycoplasma pneumoniae patient samples, United States, January – December 2023.

ICD10 code	Description
A49.9	Bacterial infection of unspecified site
B27.99	Infectious mononucleosis
H60	Diseases of the ear and mastoid process
H65	Acute serous otitis media
H66.002	Otitis media
H92.03	Otalgia
J00	Acute upper respiratory infections
J01.90	Acute sinusitis
J02.9	Acute pharyngitis
J03.90	Acute tonsillitis
J05	Acute obstructive laryngitis (croup)
J06.9	Acute upper respiratory infections of unspecified site
J15.4	Bacterial pneumonia
J15.9	Bacterial pneumonia, unspecified
J18.9	Pneumonia
J20.9	Acute bronchitis
J40	Bronchitis
R05.1	Acute cough
R05.9	Unspecified cough
R06.02	Abnormal breathing, wheezing
R21	Rash and other nonspecific skin eruption
R50.9	Fever, unspecified

The ICD-10 code J06.9 (Acute upper respiratory infection) was associated with 163 (21.5%) of the total *M. pneumoniae* positive specimens. The second most frequent ICD-10 code associated with 137 (18%) positive specimens was J02.9 (Pharyngitis). The ICD-10 codes R50.9 (Elevated body temperature/Fever) and R05.1 (Acute cough) were associated with 370 (58.35%) of the *M. pneumoniae* positive samples. Beginning from August 2023, ICD-10 codes associated with otitis media (H66) were also reported in a total of 23 (3%) patients, mostly in the 0-10- and 11-20 years age groups. Within the same age groups, 28 (3.6%) patients positive for *M. pneumoniae* had the ICD-10 code J00 (Nasopharyngitis) exclusively reported that was not observed in other age groups.

## Discussion

Beginning with the early reports of increased “walking pneumonia” cases in China during 2023 [7, 8], there has been a global resurgence in the outbreaks of *M. pneumoniae* infections, especially from July onwards [5]. Between October and December 2023, Nordholm et al. demonstrated that Denmark reported a marked increase in respiratory disease caused by *M. pneumoniae* with the highest proportion of the positive cases (39%) being reported from the 6-12 years age group [9]. A recent Dutch study found *M. pneumoniae* to be the leading cause of recurrent RTIs in children. In addition, the study found a significant correlation between *M. pneumoniae* carriage in the family members of a child positive for *M. pneumoniae* as opposed to family members of *M. pneumoniae*-negative children [10]. Data from this study also shows a similar trend where the highest instances of *M. pneumoniae*-positive cases were observed in children and adolescents.

The COVID-19 pandemic and especially the ensuing non-pharmaceutical interventions (NPIs) placed in response to the pandemic had a dramatic impact on the circulation of various bacterial and viral respiratory pathogens. As the aerosol spread of SARS-CoV-2 became established, NPIs like mask-wearing, social distancing and personal hygiene like frequent hand washing and sanitizing were stressed upon by public health authorities around the world [11]. These measures resulted in a dramatic reduction of the overall viral pathogen load during 2021 with instances of Influenza Virus and Respiratory Syncytial Virus plummeting to almost zero in the temperate regions (FluSurv-NET; RSV-NET). Since the easing of the pandemic-related lockdown and travel restrictions, there were reports of the reemergence of various respiratory viral pathogens [12]. Although instances of *M. pneumoniae* infections decreased globally during the COVID-19 pandemic [5], a multi-center retrospective study in the United States found that *M. pneumoniae* was the highest co-infection for adult COVID-19 patients with poorer prognosis and outcomes as compared to individuals with independent SARS-CoV-2 infection [13].

Administrative electronic health records (EHR) can prove to be a very useful resource for disease surveillance and public health research [14]. We wanted to assess if the current outbreak had any specific ICD-10 diagnostic codes associated with the *M. pneumoniae*-positive population that would allow organism-level identification and help guide subsequent clinical intervention. Although we found certain ICD-10 codes specifically associated with the younger population, no significant difference was observed in the various ICD-10 codes associated with the *M. pneumoniae* positive and the population positive for other pathogens causing influenza-like illness. For example, 161 (21.2%) *M. pneumoniae-positive* samples had the ICD-10 code J06.9 associated with them which was not significantly different from 49,023 (20%) of the patients that were positive for other respiratory pathogens with the same associated ICD-10 code [*Χ*2 (1, *N*=757) = 1.27, *p* = 0.25]. ICD codes have been found to have low sensitivity but high specificity in predicting the organism-specific etiologies for pneumonia, detected via laboratory testing, suggesting that the use of ICD codes may underestimate the prevalence of certain pathogens in the surveillance of syndromic infections like pneumonia [15].

In a multicenter, prospective EPIC study, *M. pneumoniae* was found to be the most common bacterial pathogen responsible for CAP among children (≥5 years). Approximately 10% of the *M. pneumoniae*-positive patients had to be admitted into the ICU; however, the infection could not be distinguished from other etiologies based on the symptoms and radiographic diagnosis. It was concluded that due to the non-specific manifestation, *M. pneumoniae* should be included in the routine diagnostic screening of children (<18 years) hospitalized with CAP [16].

**Figure 2:**
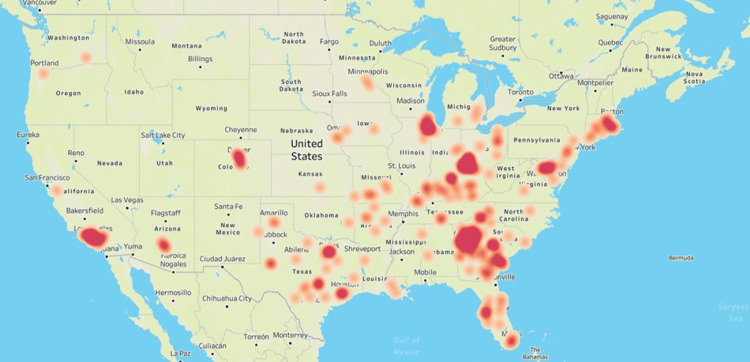
Geographic distribution of the Mycoplasma pneumoniae outbreak in 2023. Heat map showing the geographic distribution of PCR-positive *M. pneumoniae* cases in the United States during January – December 2023. The figure was created by the authors in Tableau (www.tableau.com) by using the zip code associated with the *M. pneumoniae*-positive patient samples submitted for respiratory pathogen testing at HealthTrackRx.

In the last three years, sometimes aseasonal, resurgence in instances of respiratory pathogen infections has been speculated to be a case of “immunity debt” caused by the COVID-19 pandemic where disruption of the regular respiratory pathogen spread resulted in low exposure levels, in turn decreasing the overall immunity against these pathogens within the population [17]. The current outbreak of this atypical bacterial pathogen could very well be a return to the usual cyclic pattern observed in the past with *M. pneumoniae* outbreaks in the United States occurring every 3-7 years [18]. Our data does not indicate that the 2023 *M. pneumoniae* outbreak is associated with any other respiratory pathogen circulating in the population, especially SARS-CoV-2 which was co-detected in only five (0.6%) of the *M. pneumoniae*-positive cases. The surveillance data presented in this study shows agreement with what is historically understood about the epidemiology of *M. pneumoniae* infections within the community [1, 19]. The 2023 *M. pneumoniae* outbreak in the outpatient population had a disproportionate representation in the younger population and appears to be concentrated in urban settings with large population concentration (Figure 2).

The present outbreak survey has several limitations. First, our surveillance data is based on the positive detection of *M. pneumoniae* using PCR, and in the absence of any serological confirmation of the infections, the "immunity debt" theory for the resurgence of the pathogen cannot be directly ruled out. Second, only the outpatient population was sampled in this study which may have introduced a bias in our surveillance data. Another shortcoming of this study is that we did not access detailed patient records or gather longitudinal data following the positive detection of *M. pneumoniae* in a patient which would have allowed further insight into the long-term impact of this outbreak.

## Conclusions

Results presented in this study serve as a snapshot of the *M. pneumoniae* infection outbreak in the outpatient population during 2023. To our knowledge, this is the first detailed survey of the outpatient population in the United States. The COVID-19 pandemic altered the seasonality and the epidemiological landscape of several respiratory pathogens including *M. pneumoniae*. Our survey data shows that the latest outbreak of *M. pneumoniae* with its higher prevalence in the younger population (0-20 years) as compared to other age groups is similar to the pre-pandemic outbreaks of the pathogen. The *M. pneumoniae* infections, during 2023, were not found to be positively correlated with other respiratory pathogens, especially SARS-CoV-2. We also found the ICD10 diagnostic codes to be highly specific in correctly establishing respiratory infections in a patient but showed lower sensitivity in identifying the causal organism. The COVID-19 pandemic underlined the gaps in our public health resources and highlighted the need for correctly identifying the respiratory pathogen to minimize infection spread and impact on the quality of life for the population. With the increased awareness of PCR as a rapid and reliable diagnostic technique for detecting both viral and bacterial pathogens, our study bolsters the continued surveillance of common and atypical respiratory pathogens as a public health measure.
